# Functional redundancy of necrotrophic effectors – consequences for exploitation for breeding

**DOI:** 10.3389/fpls.2015.00501

**Published:** 2015-07-08

**Authors:** Kar-Chun Tan, Huyen T. T. Phan, Kasia Rybak, Evan John, Yit H. Chooi, Peter S. Solomon, Richard P. Oliver

**Affiliations:** ^1^Centre for Crop Disease Management, Department of Environment and Agriculture, Curtin University, BentleyWA, Australia; ^2^Plant Sciences Division, Research School of Biology, Australian National University, CanberraACT, Australia; ^3^School of Chemistry and Biochemistry, University of Western Australia, PerthWA, Australia

**Keywords:** septoria, nodorum, stagonospora, phaeosphaeria, necrotrophic fungi, effectors

## Abstract

Necrotrophic diseases of wheat cause major losses in most wheat growing areas of world. Tan spot (caused by *Pyrenophora tritici-repentis*) and septoria nodorum blotch (SNB; *Parastagonospora nodorum*) have been shown to reduce yields by 10–20% across entire agri-ecological zones despite the application of fungicides and a heavy focus over the last 30 years on resistance breeding. Efforts by breeders to improve the resistance of cultivars has been compromised by the universal finding that resistance was quantitative and governed by multiple quantitative trait loci (QTL). Most QTL had a limited effect that was hard to measure precisely and varied significantly from site to site and season to season. The discovery of necrotrophic effectors has given breeding for disease resistance new methods and tools. In the case of tan spot in West Australia, a single effector, PtrToxA and its recogniser gene *Tsn1*, has a dominating impact in disease resistance. The delivery of ToxA to breeders has had a major impact on cultivar choice and breeding strategies. For *P. nodorum*, three effectors – SnToxA, SnTox1, and SnTox3 – have been well characterized. Unlike tan spot, no one effector has a dominating role. Genetic analysis of various mapping populations and pathogen isolates has shown that different effectors have varying impact and that epistatic interactions also occur. As a result of these factors the deployment of these effectors for SNB resistance breeding is more complex. We have deleted the three effectors in a strain of *P. nodorum* and measured effector activity and disease potential of the triple knockout mutant. The culture filtrate causes necrosis in several cultivars and the strain causes disease, albeit the overall levels are less than in the wild type. Modeling of the field disease resistance scores of cultivars from their reactions to the microbially expressed effectors SnToxA, SnTox1, and SnTox3 is significantly improved by including the response to the triple knockout mutant culture filtrate. This indicates that one or more further effectors are secreted into the culture filtrate. We conclude that the *in vitro*-secreted necrotrophic effectors explain a very large part of the disease response of wheat germplasm and that this method of resistance breeding promises to further reduce the impact of these globally significant diseases.

## Introduction

Effectors are defined as molecules that are produced by microbial pathogens that interact with specific “recognition” gene products in the plant host so as to affect the outcome of the contact – disease susceptibility or resistance (Tyler and Rouxel, 2012; Rovenich et al., 2014; Vleeshouwers and Oliver, 2014). Pathogen species are believed to harbor up to a few hundred such effectors and plant host species contain a much larger number of recognition genes to cope with the plethora of pathogens with which they will come into contact.

Plant pathogens are conventionally described as either biotrophic, in which the infected host tissue remains alive or necrotrophic, where host tissues are killed. In typical biotrophic interactions, recognition of an effector by a specific plant recognition gene (normally termed an *R*-gene) leads to a defense response and elimination of the pathogen. Recognition of just one such effector is sufficient to render the others redundant (Stotz et al., 2014). Such functional dominance is characteristic of interactions of biotrophic pathogens and accounts for the marked differential between resistance and susceptibility in these diseases.

Necrotrophic pathogens also produce effectors that induce a defense like response upon recognition by an R-gene-like recogniser protein (Lorang et al., 2007; Faris et al., 2010) but are distinguished by their ability to survive in the affected plant tissue and go on to proliferate and sporulate. Indeed, necrotrophic disease is clearly promoted by the recognition of effectors (Oliver and Solomon, 2010).

Like biotrophic pathogens, necrotrophs also produce many effectors. The question we address here is how do the multiple effector/recogniser interactions cooperate to cause disease? Necrotrophic diseases are typically quantitative in nature; the null-hypothesis is that each effector/recogniser interaction operating in a given situation acts additively to produce the degree of necrosis and that this directly translates into a corresponding level of disease. Depending on the context, the level of disease can be defined in terms of either as loss of yield or quantity of pathogen sporulation.

This question has practical importance as effector recognition has been adopted by breeders as a partial proxy for field resistance testing (Vleeshouwers and Oliver, 2014). Can the disease resistance of new cultivars be accurately predicted from the response to effectors of the input germplasm?

*Parastagonospora* (syn. ana, *Stagonospora*; teleo, *Phaeosphaeria*) *nodorum* (Berk.; Quaedvlieg, Verkley, and Crous) is the causal agent of Septoria nodorum blotch (SNB) on wheat (Solomon et al., 2006a; Quaedvlieg et al., 2013) and is responsible for significant yield losses in some areas of the world (Murray and Brennan, 2009; Oliver et al., 2012). Losses in the West Australian wheat belt amount to greater than AUD$100 m pa. Breeding for disease resistance has been a priority but has been hampered by the quantitative nature of the interaction. Wheat genetic analysis using infection assays as the phenotype has revealed a multitude of quantitative trait loci (QTL) and efforts to define molecular markers acceptable to breeders has proved frustrating (Oliver et al., 2012).

The discovery of multiple necrotrophic effectors has provided a clear framework to dissect the disease and has provided breeders with much needed tools (Friesen et al., 2006, 2008, 2009, 2012; Chu et al., 2010; Faris et al., 2011; Waters et al., 2011; Abeysekara et al., 2012; Crook et al., 2012; Liu et al., 2012; Oliver et al., 2012; Tan et al., 2012; McDonald et al., 2013). Our working hypothesis is that the disease can be explained by the interaction of effectors (which all appear to be small proteins secreted into culture media) and their corresponding recognition genes. Genetic analysis of the response to purified effectors has identified several wheat genetic loci that correspond to regions conferring susceptibility to the disease.

Thus far, three necrotrophic effector genes have been cloned from *Parastagonospora nodorum*. These are *SnToxA* (for which the recognition gene *Tsn1* has been cloned; Liu et al., 2006; Faris et al., 2010), *SnTox3* (Liu et al., 2009), and *SnTox1* (Liu et al., 2012). The corresponding recognition genes *Snn3* and *Snn1* have been mapped but not yet cloned. Furthermore, it is clear that several other effectors operate in this pathosystem (Friesen et al., 2012; Tan et al., 2014; Gao et al., 2015).

We have previously examined the degree of correlation between the effector sensitivities of current cultivars of West Australian cultivars and their reported field resistance (Tan et al., 2014). Unlike the tan spot system for which there is a clear dominance of one effector/recogniser interaction (ToxA/*Tsn1*; Moffat et al., 2014), no single effector had a similarly dominating role in SNB. One consideration was that all the current cultivars were sensitive to at least one of these three effectors SnToxA, 1 or 3.

Our overall goal is to model the disease susceptibility of wheat cultivars from knowledge of their effector sensitivities. We seek to understand the relative importance of each known effectors, formulate strategies to identify novel effectors/their corresponding host recogniser genes/QTLs and determine how they interact in the SNB interaction.

In this study, we have constructed a *P. nodorum* strain deleted in *SnToxA, SnTox1*, and *SnTox3*. This approach allows us to detect further secreted effectors and determine how recognition corresponds to disease expression without the interference of *SnToxA, SnTox1*, and *SnTox3*. We demonstrate that the removal of the three effectors reduced but did not entirely eliminate pathogenicity. We conclude that the secreted necrotrophic effector-recogniser model remains sufficient to explain the disease and provides a useful framework for cultivar resistance breeding.

## Materials and Methods

### Wheat Cultivars

All wheat cultivars used in this study were obtained from the Australian Winter Cereal Collection (Tamworth, NSW, Australia) and grown in vermiculite in a growth chamber at 21°C with a 12 h light/dark cycle for 2 weeks prior to infection or infiltration. Current SNB disease resistance ratings (DRR) of commercial cultivars were obtained from the Department of Agriculture and Food Western Australia (DAFWA; Shackley et al., 2013). For statistical purposes, a numerical scoring system was assigned to all DRR categories: (1) very susceptible; (2) susceptible–very susceptible; (3) susceptible; (4) moderately susceptible–susceptible; (5) moderately susceptible; (6) moderately resistant–moderately susceptible. Note that no cultivars are scored in categories 7 to 10.

### *SnToxA, SnTox1*, and *SnTox3* Triple Gene Deletions in *P. nodorum*

**FIGURE 1:**
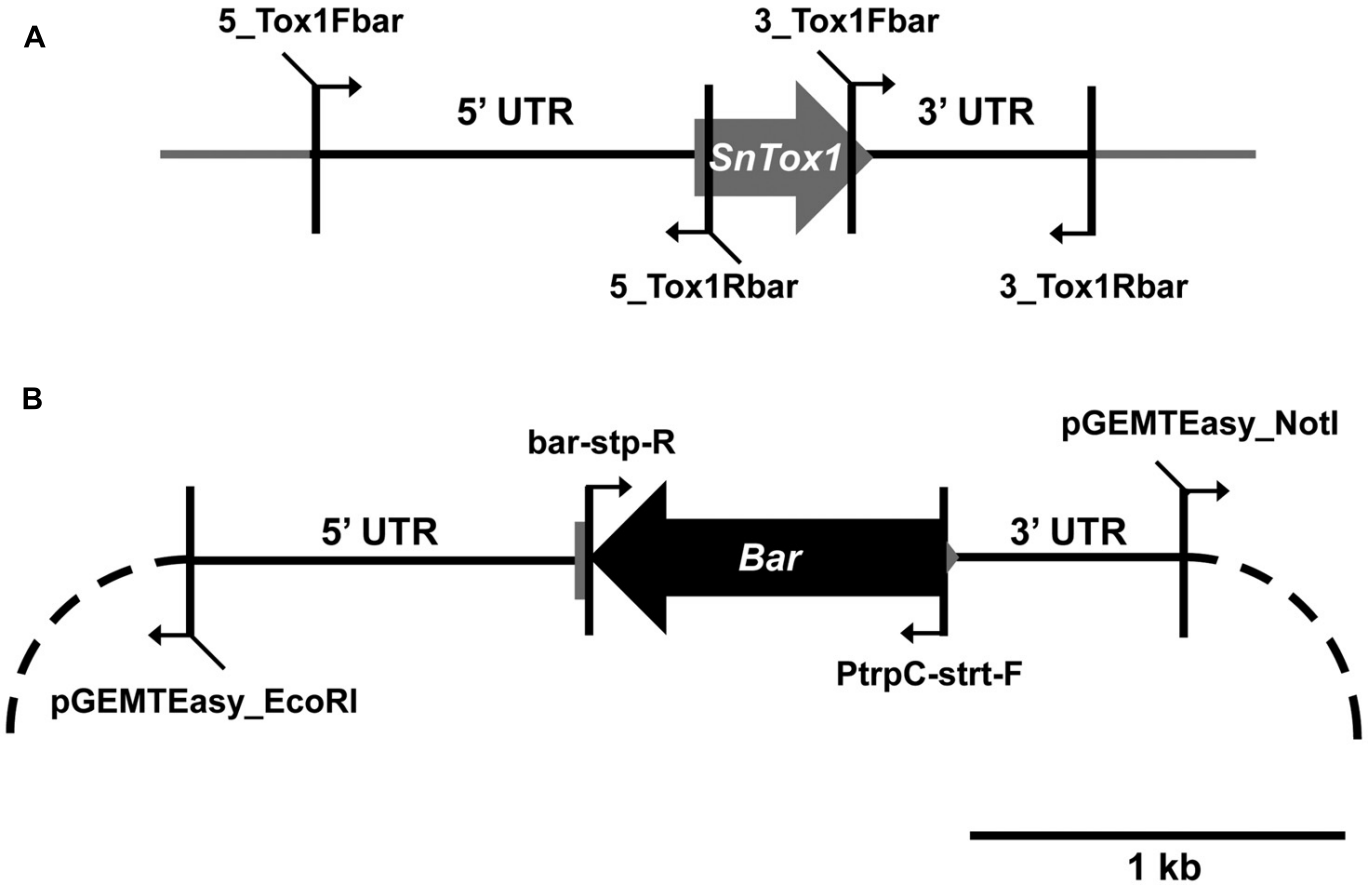
**Construction of the *SnTox1* deletion vector. (A)** 5′ and 3′ UTR of SnTox1 were amplified with primers that contain flanking sequences for *Bar*. **(B)** All four fragments were simultaneously recombined using the Gibson assembly reaction into pGEMT-Easy (dash). The resulting vector containing the *SnTox1-Bar* knockout construct was PCR-amplified with 5_Tox1Fbar and 3_Tox1Fbar for transformation in *toxa3*-10 to result in *SnToxA, SnTox1*, and *SnTox3* deletant mutants.

*Parastagonospora nodorum* SN15 strains deleted in *ToxA, SnTox1*, and *SnTox3* (*toxa13*) were created through sequential transformations using homologous-gene knockout vectors that were generated from fusion PCR (Solomon et al., 2006b) and Gibson assembly (Gibson et al., 2009; **Table 1**). All PCR amplifications were performed with Phusion *Taq* DNA polymerase (New England Biolabs, Ipswich, MA, USA). The *SnTox3* deletion construct harboring a phleomycin resistance cassette described in Tan et al. (2014) was transformed into SN15 *tox18* carrying a *SnToxA* deletion to facilitate gene knockout. PCR was used to identify the appropriate mutants deleted in *SnTox3*. A robust quantitative PCR was used to determine the integration copy number of *SnTox3* deletion constructs in all transformants (Solomon et al., 2008). The mutant *toxa3*-10 carrying a single copy *SnTox3* deletion vector insertion was subsequently selected for *SnTox1* deletion (**Supplementary Figure S1**). The *SnTox1* deletion vector was constructed using the Gibson assembly mix (New England Biolabs, Ipswich, MA, USA). The 5′ and 3′ UTR regions of SnTox1 were PCR amplified from genomic DNA using 5_Tox1Fbar, 5_Tox1Rbar, 3_Tox1Fbar, and 3_Tox1Rbar. Both flanking regions were simultaneously fused to the *Bar* gene (phosphinothricin acetyl transferase) derived from pBARKS1 (obtained from Fungal Genetics Stock Center) and pGEMT-Easy for propagation (**Table 1**; **Figure 1**). The resulting gene knockout vector was PCR-amplified for transformation to facilitate gene knockout according to Solomon et al. (2004) with modifications. Glufosinate was extracted from commercial herbicide Basta containing 200 g.l^-1^ glufosinate ammonium (Bayer Cropscience, Monheim, Germany) using chloroform as described previously (Nayak et al., 2006). An equal volume of chloroform was mixed vigorously with the Basta herbicide and centrifuged at 6,000*g* for 30 min. The upper aqueous layer containing glufosinate was retained. *Bar* transformants were selected on minimal medium containing 13 mM NH_4_Cl as the sole nitrogen source and 8 μl.ml^-1^ of extracted glufosinate. PCR was used to identify the appropriate mutants deleted in *SnTox1*. Quantitative PCR was used to determine the integration copy number of *SnTox1* deletion constructs in all transformants according to Solomon et al. (2008) with modifications. 5_Tox1qPCRF (5′-CGTAAAGAGCCGAAGATATGCC-3′) and 5_Tox1qPCRR (5′- ATAGCCCAACAGATAGGCCC-3′) were used to amplify 123 bp of the 5′ UTR homologous region immediately adjacent to the *Bar* marker of the KO cassette. Wild-type *SnTox1* was as a standard control for copy number determination of the *SnTox1* knockout cassette in the mutants. All fungal strains used in this study were described in **Table 2**.

**Table 1:** Primers used to construct the *SnTox1* deletion vector.

Primer	Sequence (5′ – 3′)
5_Tox1Fbar	CCGCAAGCAGATACAGCCGA
5_Tox1Rbar	**TGCCCGTCACCGAGATTTAG** GCCTAAGCCCTCAAACGTGAG
3_Tox1Fbar	**TCAATATCATCTTCTGTCGAC** AACCCTTGCACCGCTGGACTAG
3_Tox1Rbar	GATTGAGGGTGAGGGGCGGG
Bar-strp-R	CTAAATCTCGGTGACGGGCA
PtrpC-strt-F	GTCGACAGAAGATGATATTGA
pGEMTEasy_EcoRI_Tox1	**TCGGCTGTATCTGCTTGCGG** GAATTCCCGCGGCCGCCATGGC
pGEMTEasy_NotI_Tox1	**CCCGCCCCTCACCCTCAATC** GCGGCCGCCTGCAGGTCGAC

*The bold text refers to overlapping sequences required to facilitate appropriate recombination of DNA fragments during Gibson assembly.*

**Table 2:** *Parastagonospora nodorum* strains used in this study.

Strain	Description	Source
SN15	Wildtype	Department of agriculture WA
*toxa-18*	SN15 deleted in *SnToxA*	Friesen et al. (2006)
*toxa3-10*	*toxa-18* deleted in *SnTox3*	This study
*toxa13-6*	*toxa3-10* deleted in *SnTox1*	This study

### Production of Necrotrophic Effectors and Infiltration

Necrotrophic effectors were produced from growth in Fries 3 medium broth as described in Liu et al. (2004). Culture filtrate containing effectors were sequentially filtered gauze, miracloth, Whatman paper, and 0.22 μm sterilizers prior to infiltration with a needleless 1 cc syringe. The necrosis reaction was scored at 10 days according to visual score of 0 to 3 as previously described (Waters et al., 2011). A score of 0 indicates insensitivity (no reaction); 1, slight chlorosis; 2, extensive chlorosis; and 3, necrosis. Varieties that scored 1 were considered weakly sensitive, whereas those that scored 2 or 3 were considered highly sensitive to the effector preparation. Infiltration assays were performed on the first leaf of 2-week old seedlings.

### Whole Plant Infection Assay

Whole plant infection assay was performed on 2 week old wheat seedlings as described (Solomon et al., 2005). Briefly, 2 week old wheat seedlings were sprayed with 1 × 10^6^ pycnidiospores suspended with 0.5% w/v gelatin using an air brush system. To facilitate the infection process, all seedlings were covered for 2 days to increase humidity. After this, plants were uncovered and the infection process was allowed to continue for an additional 5 days prior the assessment of the disease symptom. A score of 1 indicates no disease symptoms were observed and a score of 9 indicates a fully necrotised plant.

### Statistical Analyses

Statistical analysis was performed using JMP 10.0.0 (SAS Institute, Cary, NC, USA) using a 2 × 2 Pearson’s chi-square test was used to test effector sensitivity datasets and SNB DRR for evidence of correlation (Tan et al., 2014). As Chi-square analyses on individual effector sensitivity scores vesus SNB DRR classes resulted in expected values less than one. As such, combining classes was used to overcome this problem on a 2 × 2 Pearson’s chi-square test (Tan et al., 2014). This approach enables a significant association to be demonstrated between SNB DRR and effector insensitivity. Crude culture filtrate sensitivity scores of 2 and 3 were pooled and scores of 0 and 1 were similarly pooled. As previously described in Tan et al. (2014), wheat cultivars that carry SNB DRR of 5 and 6 were pooled separately from scores 1 to 4. Cultivars with mixed effector sensitivity were treated as missing values by the statistical software.

## Results

### Deletion of SnToxA, SnTox1, and SnTox3 in *P. nodorum* SN15

SN15 is an aggressive *P. nodorum* wildtype isolate that carries *SnToxA, SnTox3*, and *SnTox1* (Hane et al., 2007; Syme et al., 2013). To develop a strain deleted for all three genes, we sequentially removed *SnTox3* and *SnTox1* from *P. nodorum tox18*, a SN15 transformant deleted in *SnToxA* (Friesen et al., 2006). Using the previously described *SnTox3*-knockout vector (Tan et al., 2014), we were able to generate 14 phleomycin resistant transformants, of which two carried the desired *SnTox3* deletion as determined by PCR. This represents 14.3% homologous recombination efficiency. We then selected *toxa3*-10 for *SnTox1* deletion. The transformation yielded 27 glufosinate-resistant transformants, of which five contained the desired *SnTox1* deletion. This represents 18.5% homologous recombination efficiency. We then selected four *SnTox1* knockout strains from the *toxa3-10* background to analyse for insert copy number (**Supplementary Figure S2**). All strains possess a single integration of the *SnTox1* knockout*-Bar* cassette at the *SnTox1* locus. From here, *toxa13-6* was selected for subsequent analyses.

### Characterization of *P. nodorum toxa13-6*

*Parastagonospora nodorum toxa13-6* was tested for its ability to produce SnToxA, SnTox1, and SnTox3 *in vitro*. Culture filtrate of the mutant was infiltrated into wheat cultivars BG261 (*Tsn1, snn1*, and *snn3*), Chinese Spring (*tsn1, Snn1*, and *snn3*), and BG220 (*tsn1, snn1*, and *Snn3*). Necrotic/chlorotic symptoms that are associated with compatible effector responses were not observed confirming the deletion of the genes (data not shown).

**FIGURE 2:**
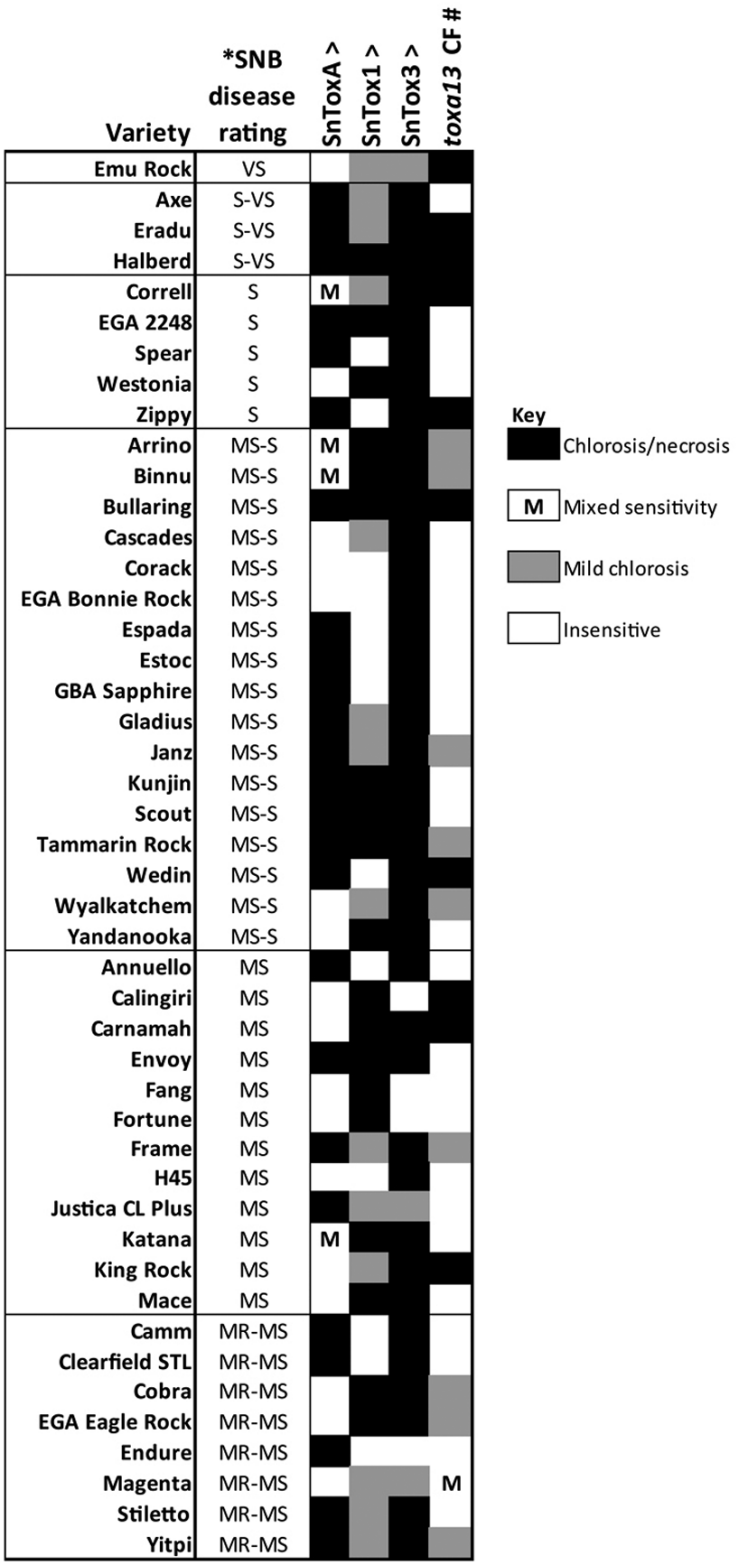
**Reactions of 46 Australian wheat cultivars to effectors [^∧^, from Tan et al. (2014)] and the *Parastagonospora nodorum toxa13-6* culture filtrate [#, this study].** ^∗^SNB disease rating was obtained from Shackley et al. (2013). VS, very susceptible; S-VS, susceptible-very susceptible; S, susceptible; MS-S, moderately susceptible-susceptible; MS, moderately susceptible; MR-MS, moderately resistant-moderately susceptible. Effector sensitivity scores are described in Supplementary Table S1.

The activity of the *toxa13-6* culture filtrate was assessed on 46 Australian commercial wheat cultivars (**Figure 2**). Ten cultivars are highly sensitive to the culture filtrate, resulting in significant chlorosis and necrosis whereas nine were mildly sensitive. It was observed that Cv. Magenta segregated in sensitivity to the culture filtrate.

**FIGURE 3:**
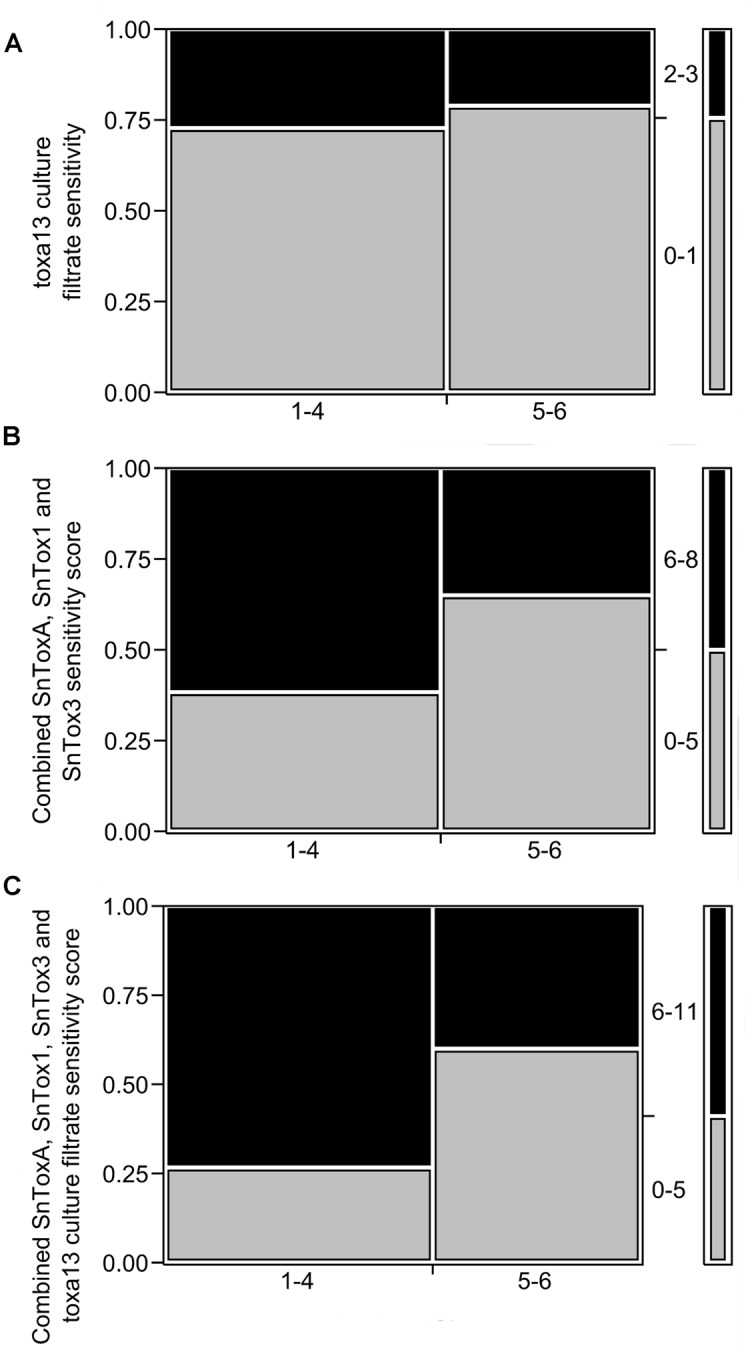
**Relationship between the distribution of 2013 septoria nodorum blotch (SNB) disease resistance rating (DRR) and reactions to **(A)***toxa13-6* culture filtrate (*p* = 0.6553); **(B)** combined SnToxA, SnTox1, and SnTox3 sensitivity scores (*p* = 0.0743); and **(C)** combined SnToxA, SnTox1, SnTox3, and *toxa13-6* culture filtrate sensitivity scores (*p* = 0.0239).** The y-axis represents the proportion of effector sensitivity for each DRR score designated on the x-axis. The right column demonstrates the distribution of effector sensitivity scores. Effector sensitivity scores are described in Supplementary Table S1. Statistical analysis was performed using frequency counts in 2 × 2 mosaic plots. The Pearson’s chi-square test was set at a significance threshold of *p* ≤ 0.05 as previously described (Tan et al., 2014).

The triple deletion of *SnToxA, SnTox1*, and *SnTox3* evidently produces further *n* necrosis-inducing factors. We then compared *toxa13-6* culture filtrate sensitivity and SNB DRR using frequency counts in a 2 × 2 mosaic plot (**Figure 3A**). No significant correlation was observed between *toxa13-6* culture filtrate sensitivity and SNB DRR (*p* = 0.6508). The combined SnToxA, SnTox1, and SnTox3 sensitivity scores correlated with the variety DRRs correlated marginally above the *p* = 0.05 significance threshold (**Figure 3B**). However, when *toxa13-6* culture filtrate scores were combined with the SnToxA, SnTox1, and SnTox3 sensitivity scores of each wheat variety (**Figure 3C**), a strongly significant correlation was observed with the SNB DRR (*p* = 0.0239). This indicates that novel necrosis inducing factors in the *toxa13-6* culture filtrate positively contribute to the severity of SNB.

**FIGURE 4:**
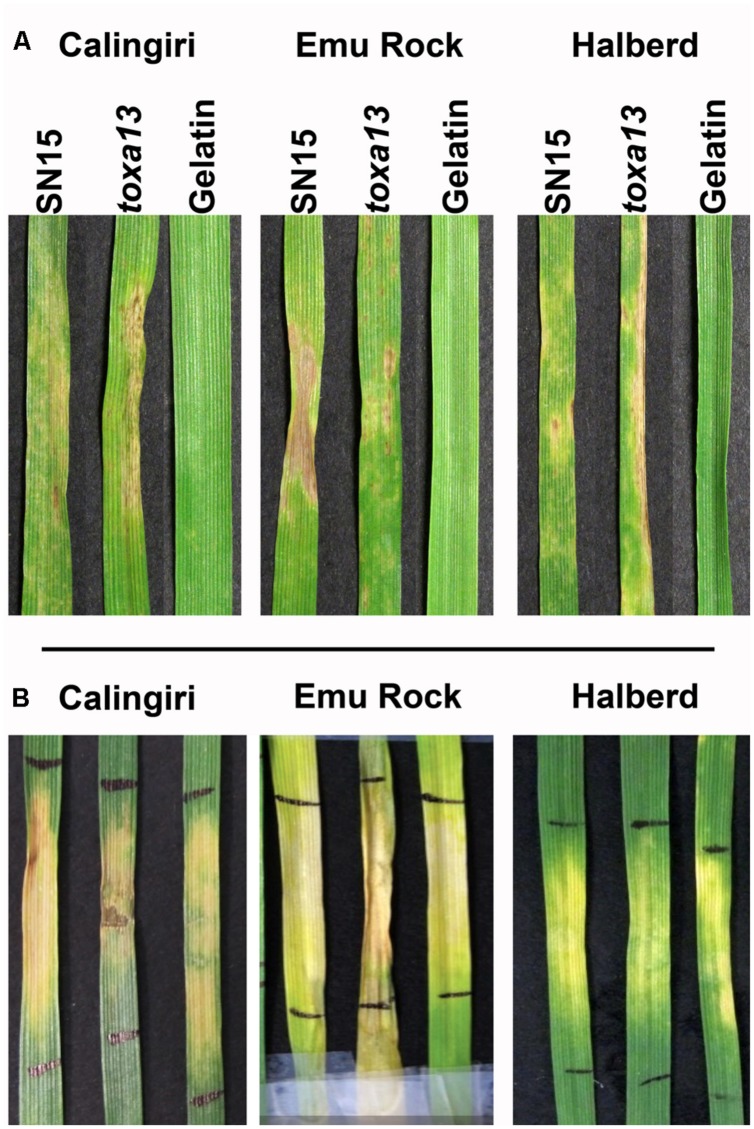
**Septoria nodorum blotch and culture filtrate symptoms of *P. nodorum toxa13-6* on Calingiri, Emu Rock, and Halberd. (A)** *P. nodorum toxa13* remained pathogenic on Calingiri, Emu Rock, and Halberd. **(B)** Distinct chlorotic and necrotic symptoms were observed after 10 days post infiltration with *P. nodorum toxa13-6* culture filtrate.

The ability of *toxa13-6* to infect wheat was assessed using a whole plant spray on selected wheat cultivars that are highly sensitive to the culture filtrate. It was observed *P. nodorum toxa13-6* can infect Calingiri, Emu Rock, and Halberd similarly to the wildtype SN15 (**Figure 4**). The other four *P. nodorum toxa13* mutants produced chlorosis/necrosis-inducing culture filtrates and remained infective on Calingiri, Emu Rock, and Halberd (data not shown).

## Discussion

Classical genetic studies indicate that SNB resistance in wheat is a polygenic trait (Wicki et al., 1999; Czembor et al., 2003). Research since 2001 (reviewed in Oliver et al., 2012) has shown that the SNB interaction involves a complex interplay of fungal effector and host dominant susceptibility genes that are necessary and sufficient to explain the polygenic and quantitative nature of the interaction. Most wheat varieties are sensitive to more than one effector and most pathogen isolates produce more than one effector.

All wheat cultivars used in this study are sensitive to one or more known effectors. This hinders the discovery of novel effector discovery through the use of *P. nodorum* culture filtrate infiltration. Furthermore, the presence of multiple QTLs which could due to the presence of multiple effector/receptor interactions can make the study of a targeted single interaction difficult as other interactions may introduce bias that mask its effect. Therefore, positional gene cloning may proof impossible under these circumstance. To overcome these difficulties, we developed pathogenic *P. nodorum* strains that are deleted in *SnToxA, SnTox1*, and *SnTox3* as a tool to discover novel effectors and SNB/sensitivity QTLs in wheat that were previously masked or unassigned. We have achieved this through the use of selectable marker genes that confer resistance to hygromycin (Solomon et al., 2004; Oliver et al., 2012) and phleomycin (Tan et al., 2008). In this study, we have implemented *Bar* as a third selectable marker for *P. nodorum* transformation. *Bar* has been adapted for use as a selectable marker in other fungal system (Avalos et al., 1989; Nayak et al., 2006; Chooi et al., 2010). Nourseothricin and G418 were tested on *P. nodorum* SN15 as potential antibiotics for fungal transformation using their respective selectable marker genes. However, *P. nodorum* showed a high level of natural tolerance to these antibiotics and cannot be used for the development of transgene resistance.

The acquisition of the triple effector knockout strain is a tool that can be used to assess the presence of further effectors relevant to commercially important wheat cultivars. These novel effectors can then be identified using biochemical separation methods. In addition, their role in virulence will be assessed through the generation of gene deletion mutants. This approach will require the deletion of additional genes in the *P. nodorum toxa13* background. To overcome limitations in marker-based selection, a selectable marker recycling system using *Cre-loxP* recombination is being developed and adapted for functional gene analysis in *P. nodorum* (Mizutani et al., 2012). Novel effectors that are verified for their role in the establishment of SNB can be implemented as a tool in resistance breeding (Vleeshouwers and Oliver, 2014).

A broad correlation between disease severity and the additive effect of effectors was observed (**Figure 4**). Ten wheat varieties showed strong sensitivity reactions to the *toxa13-6* culture filtrate. We also demonstrated that three of these wheat cultivars are highly susceptible to SNB caused by the mutant. This clearly indicates that the major effectors are secreted and function as disease determinants (**Figure 3**). This approach will enable the selection of wheat cultivars that show differential sensitivity to the *P. nodorum toxa13* culture filtrate and the construction of wheat mapping populations to genetically identify novel QTLs that confer effector sensitivity and disease susceptibility/resistance. From here, reliable genetic markers that are closely linked with QTLs of interest will be identified and used as a tool in resistance breeding in parallel with effectors to facilitated the ultimate removal of dominant sensitivity traits in wheat (Oliver et al., 2013; Vleeshouwers and Oliver, 2014).

The response of cultivars to necrosis and chlorosis-inducing effectors in the culture filtrate secretome can be compared to the field responses. The correlation between effector sensitivity and DRR is complex. We showed previously that there a significant correlation to SnTox3 (Tan et al., 2014) using DRR data available at that time. Here we show that the best correlation with the current DRR is when reactions to the three cloned effectors plus the culture filtrate from the triple mutants are combined. The correlation is significant (**Figure 3B**) and so this indicates that breeding by selecting germplasm that is insensitive to the three effectors and the culture remains a viable strategy but that functional redundancy exists between effectors. Purification of the effectors and their individual use should improve the correlation still further.

Nonetheless, whilst the correlation is significant, it is not a simple additive reaction. Epistatic effects have been observed, whereby SnToxA or SnTox1 sensitivity has been found to eliminate the reaction to SnTox3 (Oliver et al., 2012). Different effectors have different effector activity, different variants of effectors have different effector activity and different recogniser genes have different responses (Tan et al., 2012). This indicates that whilst elimination of effector sensitivities from breeding programs will lead ultimately to improved resistance, individual steps may have an impact that is too small to be noticeable or indeed may be zero. Conversely, the impact of elimination of effector sensitivities is predicted never to be negative. Trade-offs in disease resistance between resistance and susceptibility have been found in the case of some genes conferring resistance to biotrophic pathogens, such effects have not yet been found for necrotrophic effectors sensitivities (Oliver et al., 2013). Whilst we need to be vigilant for cases where the elimination of effector sensitivities has a negative pleotropic effect, the current necrotrophic effector model is both necessary and sufficient to explain all that is known about these disease interactions and to inform strategies for disease resistance.

## Conflict of Interest Statement

The authors declare that the research was conducted in the absence of any commercial or financial relationships that could be construed as a potential conflict of interest.

## Supplementary Material

Figure S1 **Copy number of the SnTox3-phleomycin resistance gene knockout cassette normalised to a single copy of actin (Act1).** The experiment was performed in biological triplicates. Standard error bars are shown.

Figure S2 **Copy number of the SnTox1-glufosinate resistance gene knockout cassette normalised to a single copy of actin (Act1).** The experiment was performed in biological triplicates. The background strain toxa3-10 was included as a single copy control. The toxa13-2 ectopic mutant was included as a 5’ UTR SnTox1 multicopy control. Standard error bars are shown.
