# Supplementary material for: Functional redundancy of necrotrophic effectors – consequences for exploitation for breeding
Source: Front Plant Sci. 2015 Jul 8;6:501. doi: 10.3389/fpls.2015.00501 (PMC4495316; doi:10.3389/fpls.2015.00501)
Supplement: Figure S1 — Copy number of the SnTox3-phleomycin resistance gene knockout cassette normalised to a single copy of actin (Act1). The experiment was performed in biological triplicates. Standard error bars are shown. [file Table_1.DOCX]

**Table S1.** Reactions of wheat cultivars to effector and fungal culture filtrate preparations.

| **Variety** | **SNB DRR** | **SnToxA^A^** | **SnTox1^A^** | **SnTox3^A^** | ***toxa13* CF^B^** | **Combined SnToxA, SnTox1, SnTox3 and *toxa13*** | **Combined SnToxA, SnTox1 and SnTox3** |
| --- | --- | --- | --- | --- | --- | --- | --- |
| **Camm** | 6 | 3 | 0 | 3 | 0 | 6 | 6 |
| **Clearfield STL** | 6 | 3 | 0 | 3 | 0 | 6 | 6 |
| **Cobra** | 6 | 0 | 2 | 2 | 1 | 5 | 4 |
| **EGA Eagle Rock** | 6 | 0 | 2 | 2 | 1 | 5 | 4 |
| **Endure** | 6 | 3 | 0 | 0 | 0 | 3 | 3 |
| **Magenta** | 6 | 0 | 1 | 1 | 3/0 | 2 | 2 |
| **Stiletto** | 6 | 3 | 1 | 2 | 0 | 6 | 6 |
| **Yitpi** | 6 | 3 | 1 | 3 | 1 | 8 | 7 |
| **Annuello** | 5 | 3 | 0 | 3 | 0 | 6 | 6 |
| **Calingiri** | 5 | 0 | 3 | 0 | 2 | 5 | 3 |
| **Carnamah** | 5 | 0 | 2 | 3 | 2 | 7 | 5 |
| **Envoy** | 5 | 3 | 2 | 2 | 0 | 7 | 7 |
| **Fang** | 5 | 0 | 2 | 0 | 0 | 2 | 2 |
| **Fortune** | 5 | 0 | 3 | 0 | 0 | 3 | 3 |
| **Frame** | 5 | 3 | 1 | 3 | 1 | 8 | 7 |
| **H45** | 5 | 0 | 0 | 3 | 0 | 3 | 3 |
| **Justica CL Plus** | 5 | 3 | 1 | 1 | 0 | 5 | 5 |
| **Katana** | 5 | 3/0 | 2 | 2 | 0 | 4 | 4 |
| **King Rock** | 5 | 0 | 1 | 2 | 2 | 5 | 3 |
| **Mace** | 5 | 0 | 2 | 3 | 0 | 5 | 5 |
| **Arrino** | 4 | 3/0 | 2 | 3 | 1 | 6 | 5 |
| **Binnu** | 4 | 3/0 | 2 | 3 | 1 | 6 | 5 |
| **Bullaring** | 4 | 3 | 2 | 3 | 3 | 11 | 8 |
| **Cascades** | 4 | 0 | 1 | 3 | 0 | 4 | 4 |
| **Corack** | 4 | 0 | 0 | 2 | 0 | 2 | 2 |
| **EGA Bonnie Rock** | 4 | 0 | 0 | 3 | 0 | 3 | 3 |
| **Espada** | 4 | 3 | 0 | 3 | 0 | 6 | 6 |
| **Estoc** | 4 | 3 | 0 | 2 | 0 | 5 | 5 |
| **GBA Sapphire** | 4 | 3 | 0 | 3 | 0 | 6 | 6 |
| **Gladius** | 4 | 3 | 1 | 3 | 0 | 7 | 7 |
| **Janz** | 4 | 3 | 1 | 3 | 1 | 8 | 7 |
| **Kunjin** | 4 | 3 | 2 | 3 | 0 | 8 | 8 |
| **Scout** | 4 | 3 | 2 | 3 | 0 | 8 | 8 |
| **Tammarin Rock** | 4 | 3 | 2 | 3 | 1 | 9 | 8 |
| **Wedin** | 4 | 3 | 0 | 3 | 2 | 8 | 6 |
| **Wyalkatchem** | 4 | 0 | 1 | 3 | 1 | 5 | 4 |
| **Yandanooka** | 4 | 0 | 3 | 3 | 0 | 6 | 6 |
| **Correll** | 3 | 3/0 | 1 | 3 | 2 | 6 | 4 |
| **EGA 2248** | 3 | 3 | 2 | 3 | 0 | 8 | 8 |
| **Spear** | 3 | 3 | 0 | 2 | 0 | 5 | 5 |
| **Westonia** | 3 | 0 | 3 | 3 | 0 | 6 | 6 |
| **Zippy** | 3 | 3 | 0 | 3 | 2 | 8 | 6 |
| **Axe** | 2 | 3 | 1 | 3 | 0 | 7 | 7 |
| **Eradu** | 2 | 3 | 1 | 3 | 2 | 9 | 7 |
| **Halberd** | 2 | 3 | 2 | 3 | 2 | 10 | 8 |
| **Emu Rock** | 1 | 0 | 1 | 1 | 2 | 4 | 2 |

^A^From Tan et al. ([2014](#_ENREF_41)), Oliver et al. ([2009](#_ENREF_27)) and Waters et al. ([2011](#_ENREF_45)).

^B^This study.
